# Factors associated with reporting behaviour of alcohol use in Moshi, Tanzania

**DOI:** 10.1192/bjo.2026.11010

**Published:** 2026-04-16

**Authors:** Joao Ricardo Nickenig Vissoci, Natan Nascimento de Oliveira, Winfrida C. Mwita, Msafiri Pesambili, Kim Madundo, Brandon A. Knettel, Deena El-Gabri, Blandina Theophil Mmbaga, Catherine A. Staton

**Affiliations:** Emergency Medicine, Division of Translational Health Sciences, Duke University, Durham, North Carolina, USA; Global Emergency Medicine Innovation and Implementation (GEMINI) Research Center, Duke University, Durham, North Carolina, USA; Duke Global Health Institute, Duke University, Durham, North Carolina, USA; Nursing Graduate Program, Department of Nursing Sciences, State University of Maringá, Maringá, Brazil; Kilimanjaro School of Medicine, KCMC University, Moshi, United Republic of Tanzania; Kilimanjaro Christian Medical Centre, Moshi, United Republic of Tanzania; Muhimbili University of Health and Allied Sciences, Dar es Salaam, United Republic of Tanzania; School of Nursing, Duke University, Durham, North Carolina, USA; Kilimanjaro Clinical Research Institute, Moshi, United Republic of Tanzania

**Keywords:** Drinking behaviour, Tanzania, alcohol drinking, attitude to health

## Abstract

**Background:**

Alcohol use disorder (AUD) is one of the most common mental health disorders globally. The diagnosis of AUD typically relies on self-reporting measures for identification, and requires that patients disclose their alcohol use to a provider.

**Aims:**

To explore the factors associated with the disclosure of alcohol use to a healthcare practitioner among injury patients, considering stigma and alcohol-related consequences of disclosure as major factors.

**Method:**

In this cross-sectional study conducted in Moshi, Tanzania, we investigated factors influencing alcohol use disclosure among injury patients. Path analysis modelling explored the relationships among Alcohol Use Disorders Identification Test (AUDIT) scores, perceived alcohol stigma, Drinker Inventory of Consequences results and disclosure.

**Results:**

Among 341 injury patients, 246 reported current or past-year alcohol use, with only 10.6% having previously disclosed alcohol use to a healthcare provider. Participants who disclosed had higher median drinks per day, elevated AUDIT scores and had experienced more alcohol-related consequences. Other factors associated with disclosure included a positive alcohol test on arrival, higher drinks per day, self-report of alcohol interfering with one’s life, problems with peers, and engagement in risk behaviours. Alcohol stigma was not associated with disclosure. The relationship between disclosure and alcohol use was mediated by alcohol-related consequences.

**Conclusions:**

People who drank more and had more alcohol-related consequences were more likely to disclose their drinking to a provider. Further research is needed to understand the factors limiting disclosure.

Alcohol use disorder (AUD) is one of the most common mental health disorders globally. The World Health Organization (WHO) estimates that over 283 million people suffer with AUD.^1^ Alcohol is the leading avoidable health risk factor in sub-Saharan Africa, accounting for a substantial portion of the global burden of death and disability. In sub-Saharan Africa, drinking behaviours such as binge-drinking and alcohol use are the second highest of any region worldwide.^2,3^

## Alcohol use disorder diagnosis

The diagnosis of AUD typically relies on self-reporting measures for identification, and thus linkage to subsequent treatment requires that patients disclose their alcohol use to a provider. However, there is limited literature on the factors that impact this disclosure. Proposed factors influencing patient disclosure or non-disclosure in high-income countries include social desirability bias, relationship with the provider, minimisation of problem-drinking behaviour, fear of discrimination and gender-related differences.^4,5^ However, in low- and middle-income country (LMIC) settings, and specifically the Tanzanian context, factors that might facilitate or hinder alcohol use disclosure to a healthcare practitioner are unclear.^6,7^

Alcohol use, both binge-drinking and chronic use, has been associated with many high-risk behaviours, including crime, aggressive driving, interpersonal violence, unintentional injuries and self-inflicted injury.^8^ Thus, alcohol not only has consequences for personal health but also profound negative effects on one’s behaviour, social interactions and social environment.^9–11^ In the literature, higher Alcohol Use Disorders Identification Test (AUDIT) scores, and therefore higher harmful alcohol use behaviour, are associated with pre-treatment readiness or, in our case, disclosure of risky alcohol use behaviour to a provider.^12^ ‘Acute alcohol patients’ and those with a positive alcohol breathalyser test upon arrival at the emergency department were more likely to disclose the use of alcohol. Disclosure patterns have been linked to the transtheoretical model of change, where individuals with more problematic patterns of drinking and those who have faced consequences for their drinking are more likely to be ready to take action to reduce their drinking.^13,14^

## Stigma and reporting behaviour

Some evidence suggests that stigma and alcohol-related consequences play fundamental roles in decision-making related to alcohol use disclosure in Tanzania, and in other LMICs. In this context, stigma is defined as ‘a negative social attitude attached to a characteristic, often perceived as a mental, physical or social deficiency’.^15^ Stigma is influenced by multiple factors, such as gender and social vulnerability.^16,17^ On the other hand, alcohol-related consequences tend to highlight the negative side of AUD, leading to greater disclosure of alcohol use.^16,17^

Stigma towards alcohol use can be seen in different contexts, and the public perception of people with an AUD can lead to negative labels such as ‘dangerous’, ‘immoral’ and ‘blameworthy’.^18^ Consequently, in LMICs, most people with AUD remain untreated because, if they seek help, they struggle to find trained providers capable of providing evidence-based, non-stigmatising care.^7,19^ This makes provider training in treatment approaches and stigma reduction key directions for the future.

For healthcare practitioners, understanding the factors that impact the disclosure of alcohol use can inform decision-making within health systems to adapt and provide the best support for individuals with AUD, as well as providing more reliable screening. Our study aims to explore the factors associated with the disclosure of alcohol use to a healthcare practitioner by injury patients. This research has the potential to inform the development of interventions to reduce barriers to alcohol use disclosure for our injury population in Moshi, Tanzania, and for other populations in similar low-resource settings. As *a priori* hypotheses, we believe that stigma could be a significant influence on reporting behaviour, with mental health stigma and alcohol use stigma being the most significant.

## Method

### Ethical standards

The study was approved by the Institutional Review Board of Duke University (IRB no. Pro00062061), the Ethics Committee of Kilimanjaro Christian Medical Centre (KCMC), Moshi, Tanzania (KCMC Research Ethical Clearance Certificate no. 497) and the National Institute of Medical Research, Tanzania (NIMR/HQ/R.8a/Vol. IX/2121). All participants signed both a hard copy and a digital copy of the informed consent Form. Participants who were illiterate, or minimally literate, had the Swahili consent form verbally summarised, witnessed and formally recorded by the study staff. This article was previously published as a preprint.^20^

### Study population/setting

Moshi is a city in the Kilimanjaro region of northern Tanzania, with a population of 143,799. Moshi is a regional trading city and has a large tourist industry; similarly, prior literature supports that the Moshi population, specifically commercial motorcycle riders^21^ and police officers,^22^ has a high proportion of AUD and injuries involving alcohol. Commercial motorcycle riders and police officers are also male-dominated fields,^23,24^ increasing the gender stigma around alcohol use and injuries in Tanzania.^25,26^

Kilimanjaro Christian Medical Centre, located in Moshi, is the third largest hospital in Tanzania and the referral hospital for the northern zone of the country. KCMC serves the urban and rural population of Moshi and a large surrounding region, and has a structured emergency department. The emergency department serves as an integral entry into the healthcare system for serious and acute health challenges. The emergency department is frequently the only financial and logistical option available for patients, placing it in a unique position, particularly in LMICs, to address AUD early. Current KCMC data show that 30% of patients who arrive at the emergency department for treatment of an injury had one of the following behaviours: (a) had consumed alcohol prior to their injury; (b) was considered a ‘harmful or hazardous drinker’ and (c) met the criteria for AUD.^27,28^ KCMC was therefore selected as a central location to assess general perceptions related to alcohol use in this region.

### Participant selection

We administered a survey to injury patients at KCMC to collect cross-sectional data for a provider on demographics, alcohol use, stigma, alcohol-related consequences and disclosure of alcohol use. Patients were enrolled in the study if they were seeking care at the KCMC emergency department for an acute injury. Patients who had a deteriorating condition, were not medically stable, were <18 years of age, were not able to communicate in Swahili or abstained from alcohol were excluded from the study. Patients who tested positive for alcohol by breathalyser upon arrival were approached for enrolment when they were clinically sober, as determined by the treating physician.

### Sample size

As previously mentioned, we believed that stigma could be a significant factor impacting the disclosure of alcohol use.^7^ As such, we established our sample size based on detecting a difference in stigma between those who disclosed and those who did not disclose alcohol use. We anticipated that the mean perceived alcohol stigma (PAS) score among those who disclosed their use would be 2.9, compared with 3.1 for those who did not disclose, with a 0.2 standard deviation. To detect this difference with 80% power, assuming 5% type 1 error, we needed 10 patients who disclosed – or 60 patients in total. We anticipated that this sample size would be larger than that for other variables tested due to the limited range of differentiation of the PAS scale. As such, with 60 patients we anticipated being able to evaluate for stigma as well as multiple other factors likely to be associated with reporting behaviour.

### Variables

Our primary outcome was the patient’s report of previous lifetime disclosure of alcohol use to a healthcare practitioner, which was assessed by the research team as a response of ‘yes’ to the questions ‘Have you ever consumed a drink containing alcohol?’ and ‘Did you ever in your lifetime talk to a medical doctor or other professional about your use of alcohol?’. We also collected data on demographic characteristics, alcohol use behaviours, problematic alcohol use behaviour, alcohol-related consequences and perceived stigma towards those who drink alcohol, for testing as factors associated with disclosure.

The demographic characteristics collected were self-reported gender and age. Alcohol use behaviours included alcohol status upon arrival at the hospital (measured with a breathalyser), age and context of first drink, drinking behaviours and patterns and reporting that drinking had interfered with one’s daily and social life.

Problematic alcohol use behaviour was assessed using AUDIT. AUDIT is an instrument used to assess alcohol dependence and hazardous and harmful alcohol use,^29^ comprising ten self-report items. The AUDIT score ranges from 0 to 40 and offers a cut-off score of 8 or higher for harmful or at-risk drinking, with 85% sensitivity and 89% specificity.^30,31^ Psychometric testing of AUDIT also performed well for injury patients and the general population in Tanzania, with the cut-off score of eight or higher being validated for the Tanzanian setting.^32,33^

The alcohol-adapted perceived devaluation-discrimination scale (PDD) assesses an individual’s PAS towards those that drink alcohol.^34^ Responses to the 12 questions are measured with a 6-point Likert scale ranging from strongly agree to strongly disagree.^34^ PAS was assessed as a factor score out of a confirmatory factor analysis model, reporting perceived alcohol stigma as an individual score on a scale from 0 to 100. The PDD scale has been used with both high-risk drinkers and abstainers, and high PAS scores have been correlated with poor mental health and a decreased likelihood of seeking alcohol treatment for drinkers in high-income settings.^34,35^

The Drinker Inventory of Consequences (DrInC) is a 50-item questionnaire used to measure alcohol-related consequences, gathering the consequences in 5 categories: interpersonal, physical, social, impulsive and intrapersonal. Each category is summed to provide both a ‘past 3-month’ and ‘lifetime’ measure of consequences, and can be combined for a total sum. DrInC is a measure of the negative consequences of drinking that has been shown to change and correlate with other alcohol outcome measures, including subjective well-being and psychosocial functioning.^36^

### Data collection

Two trained research nurses, each with over 10 years of experience administering surveys on sensitive topics (alcohol use, sexual history, HIV, social stigma) verbally conducted these surveys in their native Swahili between July 2016 and May 2017. The nurses underwent a week-long training in medical and research ethics, background for the tools, tool validation for Swahili language and comprehension, appropriate administration of the tools and project protocol. The research nurses screened patients in the emergency department, on their arrival and following medical stabilisation, for inclusion and exclusion criteria, administered informed consent and conducted breathalyser testing for those who met the criteria. The surveys were collected on paper in a quiet, secluded location, were checked for completeness and errors and entered into an online database with a secondary quality control performed both during and following data entry.

During the creation of the survey we performed translation and back-translation of each item, and the trained research team assessed each question for comprehension and language equivalence. Subsequently, the team conducted pilot surveys among patients and changed required questions prior to finalisation of the questionnaire.

### Data analysis

Descriptive statistics were used to report the research results: frequencies and percentages were compiled for categorical variables; medians and interquartile ranges (IQRs) were reported for numerical variables. Hypothesis tests were used to estimate significant differences between the variables. First, we tested for normality with the Kolmogorov–Smirnov test, with Lilliefors correction for the numerical variables; when criteria was not met we performed the Mann–Whitney *U*-test. For categorical variables we used Fisher’s exact test because most of the categories had a value of less than eight.

We used path analysis modelling to evaluate the complex relationships among PAS, AUDIT score and DrInC results, and their impact on disclosure of alcohol use to a healthcare provider. Models were built using a structural equation modelling approach. The model was estimated using diagonally weighted least squares, considering the non-normal characteristics of our variables’ distributions, and to estimate a logit estimation with the binary outcome (lifetime disclosure or non-disclosure of alcohol use to a healthcare provider). We also estimated odds ratios and 95% confidence intervals for interpretation of results.

The analysis was performed using the lavaan package for R Language for Statistical Computing software, version 4.2 for Windows (R Core Team, Vienna, Austria; https://cran.r-project.org/), and considered a significance level of 95% for all statistics estimated.

## Results

Of a total of 341 injury patients, 95 abstained from alcohol, leaving 246 who reported current alcohol use or alcohol use in the past year. The overall number of patients in our study who had previously disclosed alcohol use to a provider is low (26, 10.6%).

### Demographics and alcohol use behaviours

The mean age of participants was 37.7 years (s.d. 14.0), with no significant differences between those who did or did not disclose the use of alcohol. Our sample included 43 women (17.5%), none of whom had previously disclosed alcohol use to providers. Of the 203 men who participated in the study, 26 (12.8%) had disclosed their alcohol use to a provider (Table 1).

**Table 1:** Sample characteristics and associations with disclosure of alcohol use (*N* = 246). Values are mean (s.d.) for continuous variables and *n* (%) for categorical variables

Variables	Total (*N* = 246)	Disclosed alcohol use (*n* = 26)	Did not disclose alcohol use (*n* = 220)	Odds ratio (95% CI)	*P*-value
Age (years), mean (s.d.)	37.6 (14.0)	40.5 (16.0)	37.3 (13.7)	1.01 (0.97–1.05)	0.53
Women, *n* (%)	43 (17.5)	–	43 (19.5)	–	
Alcohol positive on arrival, *n* (%)	**19 (7.7)**	**5 (19.2)**	**14 (6.4)**	**3.87 (1.01–14.2)**	**0.04**
Drinks per day, median (IQR)	**2.0 (2.0–3.0)**	**4.0 (2.0–5.0)**	**2.0 (1.0–3.0)**	**1.51 (1.18–2.07)**	**<0.01**
Drinking interfered with your life, *n* (%)	**67 (27.2)**	**16 (61.5)**	**51 (23.2)**	**17.45 (4.31–118.49)**	**<0.01**
Drinking problems with social peers, *n* (%)	**75 (30.4)**	**16 (61.5)**	**59 (26.8)**	**6.86 (2.09–27.21)**	**<0.01**
Drinking and risk behaviour for injury, *n* (%)	**51 (20.7)**	**12 (46.2)**	**39 (17.7)**	**5.93 (1.72–20.89)**	**<0.01**
Drinking led to arrest, *n* (%)	12 (9.5)	4 (15.4)	8 (3.6)	2.43 (0.11–27.10)	0.48
Hazardous drinking level (if AUDIT ≥8), *n* (%)	90 (36.6)	16 (61.5)	74 (33.6)	2.43 (0.11–27.10)	0.48
AUDIT score, median (IQR)	**5.0 (2.0–12.0)**	**19.0 (6.0–21.5)**	**4.0 (2.0–8.0)**	**1.08 (1.02–1.16)**	**<0.01**
DrInC, median (IQR)	**7.0 (4.0–19.0)**	**25.0 (10.5–36.0)**	**6.0 (3.0–15.0)**	**1.10 (1.05–1.17)**	**<0.01**
Perceived alcohol stigma, median (IQR)	24.1 (12.2–43.0)	24.1 (12.9–38.4)	22.7 (12.2–43.2)	0.91 (0.43–1.90)	0.82

IQR, interquartile range; AUDIT, Alcohol Use Disorders Identification Test; DrinC, Drinker Inventory of Consequences.

Bold font indicates statistical significance.

As shown in Table 1, from our sample 7.7% tested positive for alcohol upon arrival at the hospital. Of the population that disclosed alcohol use to a provider, the median number of drinks consumed per day was 4.0 (IQR 2.0–5.0). More than a quarter of patients reported that drinking had negatively impacted their life (27.2%) and their social relationships (30.4%). Overall, 9.5% had been arrested and 20.7% reported that their drinking had influenced them to engage in risk behaviours responsible for their injuries (Table 1).

The median AUDIT score was 5.0 (IQR 2.0–12.0), and 36.6% of participants had a hazardous drinking level. Patients who reported disclosing alcohol use to a health care provider demonstrated a statistically significantly higher AUDIT score, with a median of 19.0 (IQR 6.0–21.5) (Table 1).

The median score for the DrInC questionnaire was 7.0 (IQR 4.0–19.0) in the overall sample. There was a significant difference for the count of alcohol-related complications as measured by DrInC between the two groups: those who had disclosed the use of alcohol reported higher scores (median 25.0, IQR 10.5–36.0) than those who had not (median 6.0, IQR 3.0–15.0) (Table 1).

Mean age exhibited a negligible association with alcohol disclosure (odds ratio 1.01, 95% CI 0.97–1.05). However, individuals testing positive for alcohol on arrival displayed a significantly increased odds ratio of disclosure of 3.87 (95% CI 1.01–14.2). Moreover, median drinks per day demonstrated an odds ratio of 1.51 (95% CI 1.18–2.07), emphasising the impact of drinking patterns on disclosure likelihood. Participants who reported a negative impact of alcohol on their lives exhibited a markedly elevated odds ratio of disclosure of 17.45 (95% CI 4.31–118.49), underscoring the association between life disruption and disclosure behaviour. Similar trends were observed for drinking problems with social peers (odds ratio 6.86, 95% CI 2.09–27.21) and drinking-related risk behaviour for injury (odds ratio 5.93, 95% CI 1.72–20.89) (Table 1).

Hazardous drinking demonstrated an elevated odds ratio for disclosure of 2.43 (95% CI 0.11–27.10). The AUDIT score showed a modest association (odds ratio 1.08, 95% CI 1.02–1.16), as did DrInC (odds ratio 1.10, 95% CI 1.05–1.17). PAS displayed an odds ratio of 0.91 (95% CI 0.43–1.90), with no significant association with alcohol disclosure to healthcare providers (Table 1).

### PAS

PAS, as measured by PDD, had a median score of 24.1 (IQR 12.2–43.0). For PAS levels there was no significant difference across patients who reported disclosing or not disclosing alcohol use with a health care provider (Table 1). We found an odds ratio of 0.91 (0.43–1.90), with a *P*-value of 0.82. PAS showed no significant differences between genders (*P* = 0.191).

### Factors impacting disclosure of alcohol use

**Fig. 1:**
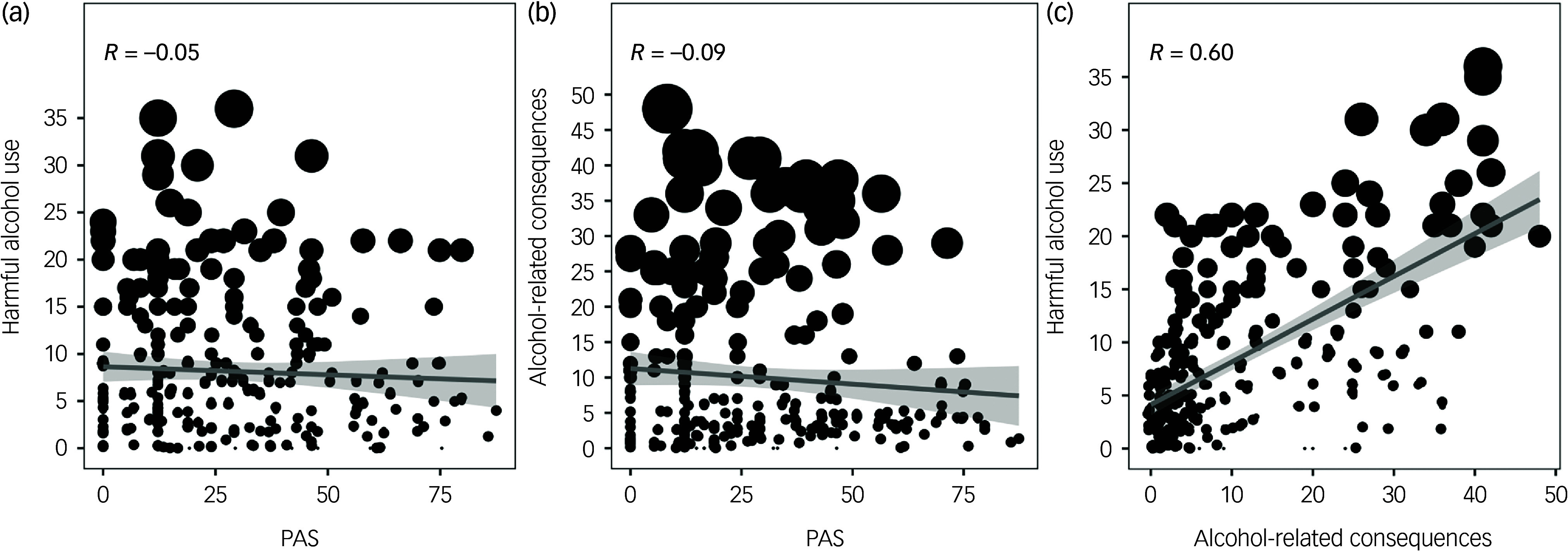
Associations between (a) harmful alcohol use and perceived alcohol stigma (PAS), (b) alcohol-related consequences and PAS and (c) harmful alcohol use and alcohol-related consequences. Harmful alcohol use is measured by the Alcohol Use Disorders Identification Test.

The level of any negative alcohol-related consequences was positively correlated with higher AUDIT scores (*R* = 0.60). PAS was associated with neither harmful alcohol use nor alcohol-related consequences (Fig. 1).

**Fig. 2:**
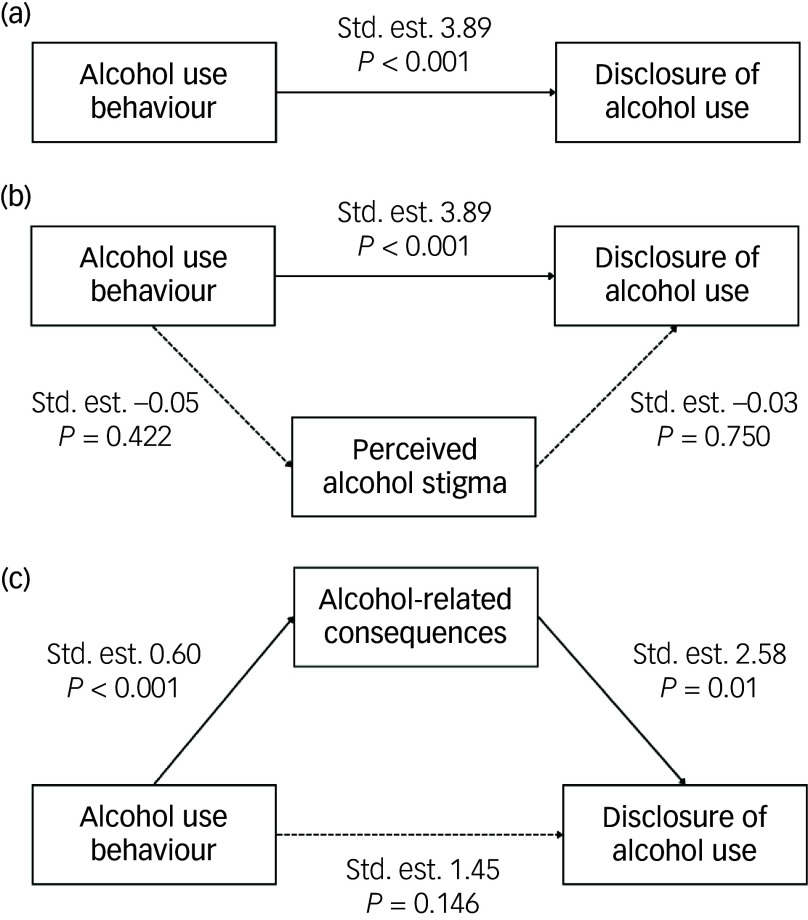
Association between harmful alcohol use and disclosure of alcohol use to healthcare providers, mediated by perceived alcohol stigma and alcohol-related consequences. Std. est., standard estimation.

The relationship between alcohol use behaviour and the disclosure of alcohol use to a healthcare professional was fully mediated by the level of alcohol-related consequences experienced by patients (Fig. 2). The indirect effect of harmful alcohol use, mediated by alcohol-related consequences, was higher (odds ratio 0.363) than the direct effect without the mediator. PAS did not mediate the association between alcohol use behaviour and disclosure of alcohol use (Fig. 2).

## Discussion

This study is among the first to explore the factors impacting the reporting behaviour of alcohol use in sub-Saharan Africa. We found that none of the women in our study disclosed alcohol use with a provider. Also, those patients who disclosed alcohol use to a provider were more likely to be alcohol-positive by breathalyser prior to injury, and to report that their drinking had an impact on their lives, social relationships and risk behaviours for injury. We found that injury patients reported high levels of perceived stigma, but there was no association between perceived stigma and disclosure of alcohol use.

Despite creating a large burden of death and disability, AUD has not been seen as a high priority in most LMICs. In addition, interventions to diminish this burden are made possible by the disclosure of alcohol use to a healthcare provider, which does not always happen due to multiple factors. The factors associated with disclosure of alcohol use to providers have not yet been studied in Tanzania, or in the broader East Africa region. To encourage accurate disclosure and diagnosis of AUD, it is imperative to describe the factors that influence disclosure. As such, our findings are of great importance to clinicians and health practitioners in the region.

It is important to acknowledge that alcohol use behaviours differ by gender.^25^ Many studies have shown that AUD is more frequent among men, although women are more likely to try to stop using alcohol.^16,25,27^ However, there are greater challenges for women to get treatment generally, which literature associates with the stigma around alcohol use among women, leading to less disclosure of this behaviour to providers and therefore diminishing access to treatment.^17,25,26^ Men in Tanzania are often expected to be the financial provider in families and to be an active participant in the community, whereas women are more often seen as caregivers; failing in these roles can lead to harsh social judgement.^37^ An in-depth analysis of gender roles and their association with alcohol-related behaviours in Tanzania is necessary to better understand alcohol use among women.

Our study found that patients with higher counts of alcohol-related consequences (DrInC) were correlated with disclosure of alcohol use to a provider. Similarly, other studies have found that high levels of drinking consequences were positively associated with patients’ perceived need for alcohol treatment.^38^ It is both advantageous and unsurprising that those who had suffered a greater alcohol-related impact on their lives disclosed alcohol to use to a professional. These data together support screening of at-risk alcohol use and consequences to facilitate referral to treatment, especially in an acute setting such as Tanzania.

Contrary to previous studies, our results from Tanzania found no association between alcohol stigma and disclosure of alcohol use. There are several potential reasons for this deviation from prior literature. First, there is a chance that stigma in Tanzania does not impact disclosure of alcohol use or care-seeking behaviour. Common barriers to care-seeking and/or disclosure are self-reliance (‘I should be strong enough to handle this’), minimising the problem (‘My drinking isn’t serious enough’), fear of social consequences, financial and structural barriers, fear of treatment and treatment pessimism.^19,39^ It is very likely that stigma does not have a strong influence compared with other common barriers. Second, cultural and translational issues may have resulted in an inappropriate understanding of our scales or questions. Although we performed rigorous translation, back-translation and team-based cognitive interviewing to ensure scale comprehension, a de novo, culturally appropriate scale could prove more sensitive to a culturally impacted topic such as alcohol-related stigma. Third, we asked patients whether they had disclosed alcohol use behaviour to a healthcare practitioner as opposed to whether they had sought care specifically for alcohol use behaviour. The perceived level of stigma and risks involved in seeking care may differ from the level associated with the act of disclosing information to a provider in the course of treatment for another issue.

### Limitations

Our study has certain limitations that should be considered when evaluating the results. First, the limited sample size has very small subgroup analyses, as seen with our small samples of women and people who disclosed their alcohol use. Our choice to estimate separate structured equation models (SEMs) for the two putative mediators was driven by concerns about model stability and over-parameterisation in a modest sample. Common rules of thumb for SEM suggest either larger absolute sample sizes (often cited as ≳200) or many observations per estimated parameter; furthermore, multi-parameter or multi-mediator models generally require substantially larger samples to produce stable estimates and narrow confidence intervals. For these reasons the present SEM results should be considered exploratory and hypothesis-generating; future studies with larger and more balanced samples should estimate combined mediator models to examine unique indirect effects while accounting for mediator intercorrelations.

Second, given our survey methodology, our results are subject to recall and social desirability bias. We attempted to limit these biases by using standardised measures; limited recall periods, including the timeline follow-back methods where appropriate; presenting the research in a compassionate and non-stigmatising manner; and assuring participants of our efforts to maintain confidentiality. Because we were asking about a patient’s prior disclosure of alcohol use, the utility of biometric information is limited. However, our patients with a positive breathalyser test were more likely to have disclosed harmful alcohol use behaviour to a provider in the past, which helped to confirm their reporting. We have specifically chosen an injury population in preparation for an intervention among this population, and therefore our data might not be generalisable to a larger, non-injury population or the general public. Cultural and language factors might also have impacted our results, but intensive translation and cultural review with our Tanzanian research team would probably have identified these issues.

In Tanzania, obstacles to timely emergency transportation to hospital result in a significant time gap between an injury and arrival at the hospital; therefore, we did not include a 6 h time limit between injury and arrival, as other WHO studies have.^40^ As such, our proportion of alcohol breathalyser-positive patients is lower than that of prior studies and international estimates,^7,17,40^ and underestimates the actual alcohol-related injury proportions of patients. This was done to be inclusive of patients with transportation issues, and to estimate the proportions of patients who might be amenable to a future intervention. Even with this limitation, having a positive alcohol breathalyser test was significantly associated with reporting behaviours.

Lastly, as the research was conducted with patients who presented at KCMC emergency department, those presenting at this health centre are most probably the ones with more serious AUDs, which could have led to a referral bias in the study sample. In this scenario the results presented show that, as the AUDIT score increases, reporting increases as well. This demonstrates that, even with this limitation, by accounting for the level of alcohol consumption and consequences, there are differences in reporting that were sensitive in this analysis.

Our study found that disclosure of alcohol use was associated with alcohol use behaviours, high AUDIT scores and high counts of alcohol-related consequences (DrInC), but not with PAS. This finding is important for healthcare professionals, especially when considering the utility of screening for AUD in emergency departments. Further research is warranted to understand the causal factors limiting disclosure of alcohol use in this setting, and the influence of stigma, which is particularly important for women who may have been reluctant to disclose any alcohol use. We also recommend universal screening for alcohol use in emergency departments, which can lead to a higher engagement with treatments and actions of health promotion; and we also highlight the need for inclusion of the emergency department as part of mental health services.

## Data Availability

De-identified data are available only upon request, in order to comply with Tanzanian ethical committee regulations.
